# Histology and Lung Nodule Fluorescence in Intraoperative Molecular Imaging With Pafolacianine

**DOI:** 10.1016/j.atssr.2024.02.003

**Published:** 2024-03-05

**Authors:** Patrick Bou-Samra, Austin Chang, Emily Guo, Feredun Azari, Gregory Kennedy, Azra Din, Taine Pechet, Doraid Jarrar, John Kucharczuk, Jarrod Predina, James Delikatny, Philip S. Low, Sunil Singhal

**Affiliations:** 1 Division of Thoracic Surgery, Department of Surgery, University of Pennsylvania Perelman School of Medicine, Philadelphia, Pennsylvania; 2 Department of Radiology, University of Pennsylvania Perelman School of Medicine, Philadelphia, Pennsylvania; 3 Department of Chemistry, Purdue University, West Lafayette, Indiana

## Abstract

**Background:**

Intraoperative molecular imaging (IMI) uses a cancer-targeted fluorescent agent injected into patients to localize tumor nodules. Pafolacianine is a folate receptor (FR)–targeted near-infrared fluorescent probe. Almost 10% of patients have false negative fluorescence findings intraoperatively. We hypothesized that tumor histology explains why lung cancer may not fluoresce.

**Methods:**

Adenocarcinoma (AC) (A549, LKR) and squamous cell carcinoma (SCC) (H127, H1264) cell lines were stained with pafolacianine. Near-infrared fluorescent microscopy was used to quantify mean fluorescence intensity. Tissue microarray slides of patients with AC and SCC were evaluated by immunohistochemistry for FR alpha (FRα) and beta (FRβ) expression. Finally, we retrospectively analyzed IMI data from clinical trials of patients with AC and SCC receiving pafolacianine.

**Results:**

AC (intensity 30.31) cell lines have a higher fluorescence intensity than SCC cell lines (intensity 5.4) (*P* < .001). On slide analysis, 93.8% of ACs expressed FRα compared with 44.4% of SCCs (*P* = .002). Finally, there were 326 patients enrolled in clinical trials: 211 had lesions localized in vivo, and 134 of these patients had pure AC or SCC. All 9 patients with SCC have a positive smoking history and a mean pack-year of 60.2 (SD 3,6), whereas 76% of patients with AC have a history of smoking and a mean pack-year of 29.3 (*P* = .02). The odds ratio for fluorescence of (AC/SCC) was 2.05 (*P* = .004) and 2.01 (*P* = .02) on univariate and multivariate logistic regression, respectively.

**Conclusions:**

During IMI with pafolacianine, a nonfluorescent nodule is more likely to be SCC than AC. AC has a high probability of fluorescing because of higher expression of FRα or FRβ, or both.

**In Short**

▪ Tumor histology appears to play in role in intraoperative molecular imaging with pafolacianine.
▪ With this folate-specific fluorophore, patients with adenocarcinoma of the lung are more likely to fluoresce when compared with patients with squamous cell carcinoma, likely attributed to increased folate expression in patients with adenocarcinoma.

Intraoperative molecular imaging (IMI) aids surgical oncologists to detect cancers during resections. Patients receive a tumor-targeted fluorescent contrast agent before surgery. This contrast agent targets cancer cells and is excited by a near-infrared (NIR) laser. An NIR camera then detects the fluorescent emission and localizes the suspicious lesion.^1^ Pafolacianine (Cytalux, OTL-38) is a fluorophore that targets the folate receptor (FR). There are 2 FRs, alpha (α)(FRα) and beta (β)(FRβ), expressed on tumor cells and tumor-associated macrophages (TAMs), respectively. A phase 2 and a phase 3 clinical trial have shown success in localizing occult disease, identifying synchronous lesions during dissection, assessing margins when evaluated on a back table, and changing the planned surgical procedure.^2^

Currently, a major challenge in IMI is the rate of false negative results. A false negative result may give surgeons confidence that a nodule is not cancer because it is not fluorescing, and this would reduce the likelihood of an R0 resection.^3^ Previous studies have shown that there is differential expression of FRα and FRβ between lung adenocarcinoma (AC) and squamous cell carcinoma (SCC) in vitro.^4^ However, those studies were limited because FRα is present on tumor cells, whereas FRβ is present on TAMs, and that is difficult to replicate in vitro. In our study, our aim was to assess whether tumor histology could be a cause of false negative lung nodules during IMI. Understanding that certain nodules that may be cancerous on imaging will not fluoresce may help surgeons make an intraoperative decision on a nonfluorescent lesion that has not been determined by biopsy to be cancer preoperatively.

The study was reviewed and accepted by the Institutional Review Board of The University of Pennsylvania. All patients have consented for participating in this research study.

## Patients and Methods

### Pafolacianine (OTL-38 or Cytalux)

Pafolacianine (chemical formula C_61_H_63_N_9_Na_4_O_17_S_4_; molecular weight, 1,414.42 Da; On Target Laboratories) is a tracer consisting of a folate analogue conjugated to the NIR fluorescent dye S0456. It is approved by the Food and Drug Administration for ovarian malignancies and recently lung cancer.^5^ Pafolacianine maximally excites at a wavelength of 774 to 776 nm and has a peak emission of 794 to 796 nm.^6^

### Evaluation of In Vitro Binding and Fluorescence Intensity of Pafolacianine by Fluorescence Microscopy

A549 is a cell line for human bronchoalveolar carcinoma, a form of adenocarcinoma. LKR is a mouse adenocarcinoma cell line. H1264 and H2170 are human squamous cell carcinoma cell lines. Post hoc image analysis was conducted with ImageJ (a public domain Java image processing program from the National Institutes of Health). Mean fluorescence intensity (MFI) was quantified by analyzing fluorescent microscopy images and measuring regions with cancer cell lines (n ≥20). Calculations were performed in triplicate.

### Assessing Folate Receptor Expression in AC/SCC Cell Lines and Human Tissue Microarrays by Immunohistochemistry

Cell lines were prepared in formalin-fixed paraffin-embedded blocks. Tissue microarray slides (TMA) (TissueArray.Com LLC) contained 34 specimens from AC and SCC. Both cell lines and TMA slides were immunostained for FRα and FRβ by using anti-FRα monoclonal antibody (Leica Biosystems, clone BN3.2) and anti-FRβ monoclonal antibody (Novus Biologicals, catalog number NBP2-43654), respectively. Once stained, specimens were imaged at 40× magnification on a Leica DM6 B microscope (Leica Microsystems). A certified pathologist manually scored specimens by using a system of 0 (no staining), 1 (<10% of carcinoma staining), 2(10%-50% of carcinoma staining), or 3 (>50% of carcinoma staining).

### Clinical Trial of Lung Cancer Intraoperative Molecular Imaging Using Pafolacianine

Our retrospective review included patients who underwent pafolacianine-guided thoracic surgery for lung lesions suggestive of malignancy at the Hospital of the University of Pennsylvania in Philadelphia between June 2015 and August 2019. The outcome of interest was in vivo localization of the suspicious lesion. Univariate logistic regression was performed, followed by a multivariate logistic regression model, to find the correlation between tumor histology and localization. Two 2×2 tables were designed to calculate the sensitivity, specificity, negative predictive value, and positive predictive value of IMI detection of both AC and SCC. Statistical analysis was performed with SPSS version 28.0 statistic software package (IBM Corp). The study was reviewed and accepted by the Institutional Review Board (IRB) of The University of Pennsylvania (IRB: 842586). All patients have consented for participating in this research study.

## Results

### Ex Vivo Comparison of OTL-38 Detection of Adenocarcinoma Compared With Squamous Cell Carcinoma

#### Pafolacianine preferentially targets adenocarcinoma cell lines

**Figure 1:**
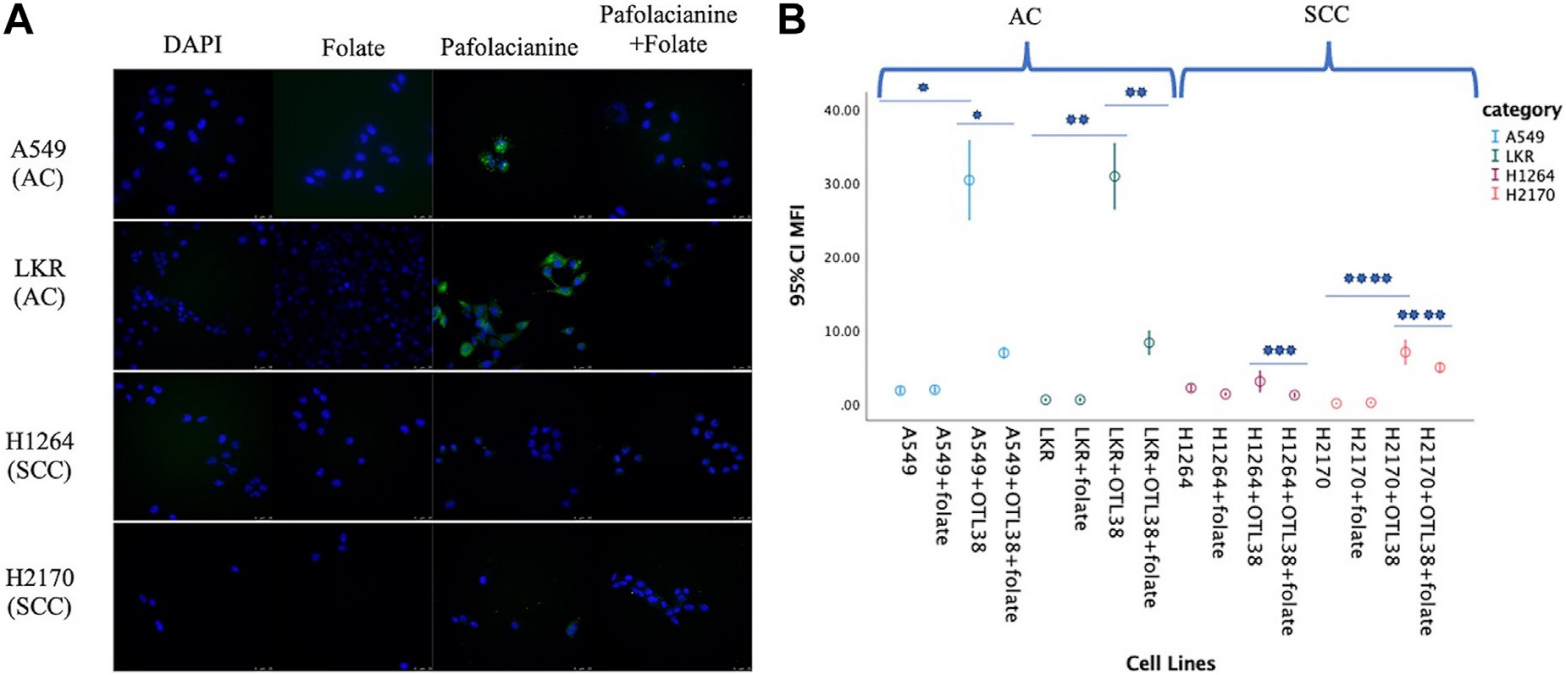
(A) Comparison of adenocarcinoma (AC) cell lines (A549 and LKR) with squamous cell carcinoma (SCC) cell lines (H1264 and H2170) when stained by pafolacianine (OTL-38). The first 2 rows are the negative control, and the fourth row is competitive inhibition. (B) Comparison of mean fluorescence intensity (MFI) among different cell lines. All images are at 40× magnification. The asterisks (∗,∗∗,∗∗∗,∗∗∗∗) denote significance at *P* < .001. DAPI, 4′,6-diamidino-2-phenylindole.

The combined MFI of both AC cell lines (A549 and LKR) when stained with OTL-38 was 30.31 AU (SD 11.3) compared with that of the SCC cell lines (H1264 and H2170), which was 5.14 AU (SD 3.8) (*P* < .001) (Figure 1).

#### Immunohistochemistry shows increased frα expression in adenocarcinoma

**Figure 2:**
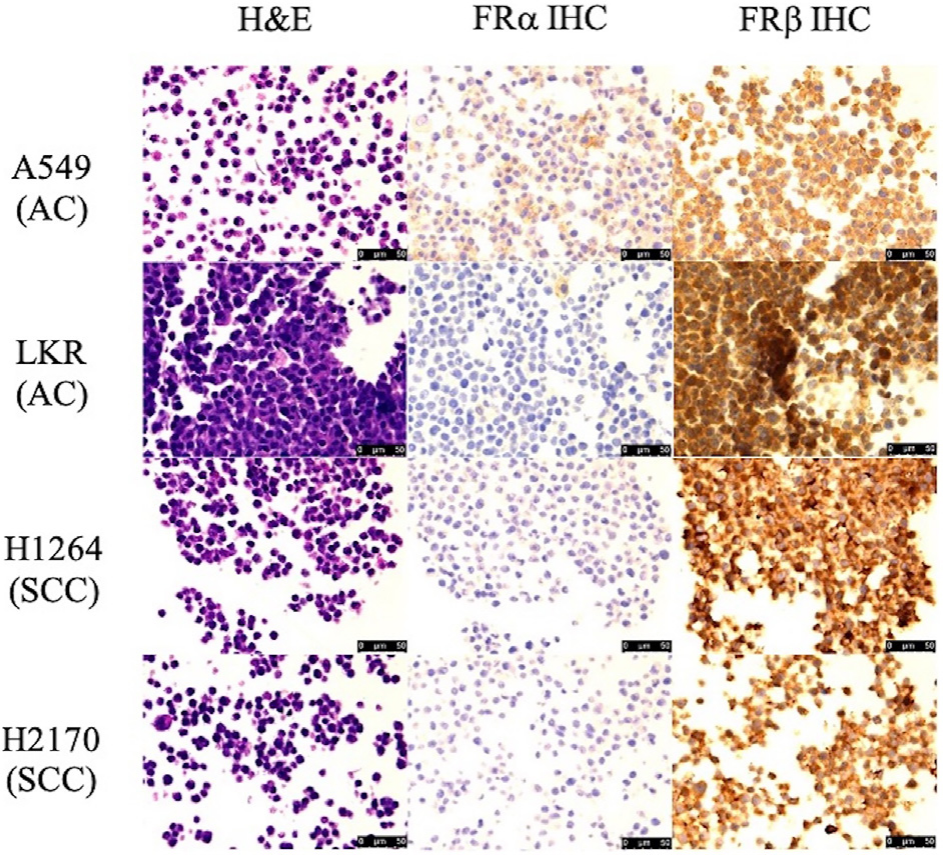
Immunohistochemistry (IHC) of adenocarcinoma (AC) and squamous cell carcinoma (SCC) cell lines for folate receptor alpha (FRα) and folate receptor beta (FRβ). The first column represents hematoxylin and eosin (H&E) staining, the second column antibodies against FRα, and the third column antibodies against FRβ. The first 2 rows represent AC cell lines, and the third and fourth row are SCC. All images are at 40× magnification. Nuclei are purple; FR stain is brown. (ACC, adenocarcinoma; SCC, squamous cell carcinoma.)

On evaluation of the cell lines previously described, we noted more FRα in the AC cell lines compared with the SCC. There was no obvious FRβ expression in either cell line (Figure 2).

**Figure 3:**
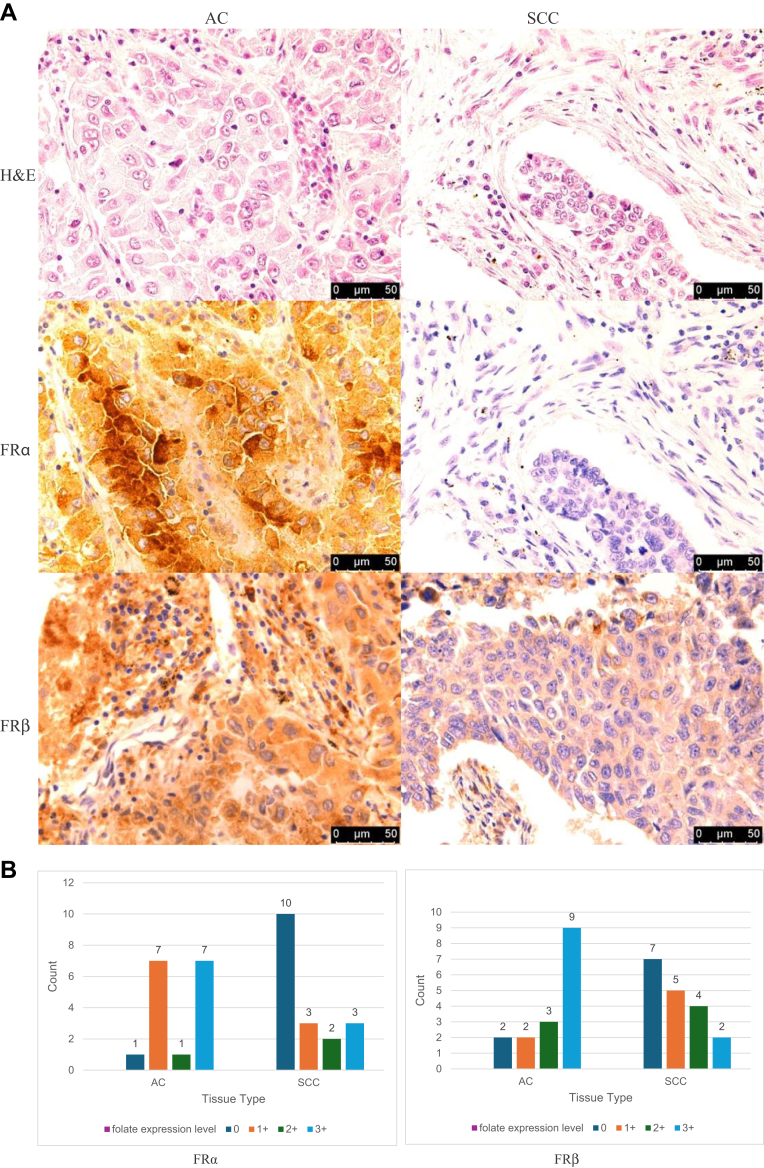
(A) Comparison of tissue microassay (TMA) slides of adenocarcinoma (AC) and squamous cell carcinoma (SCC) using immunohistochemistry (IHC). (First row) Hematoxylin and eosin (H&E) sections of AC and SCC. (Second row) (left) AC and (right) SCC TMA section stained with antibody against folate receptor alpha (FRα). (Third row) (left) AC and (right) SCC TMA section stained with antibody against folate receptor beta (FRβ). (B) Distribution of FRα and FRβ expression in both AC and SCC.

We examined 34 slices on a single TMA slide of AC and SCC tissues (Figure 3A, 3B). There were 16 AC samples and 18 SCC samples. The TMA slices of AC demonstrated FRα expression greater than 0 in 93.8% (15 of 16) of cases, whereas only 44.4% (8 of 18) of SCC samples exhibited such expression (*P* = .002). When it comes to FRβ expression, it was observed in 87.5% (14 of 16) of patients with AC and in 61.1% (11 of 18) of patients with SCC (*P* = .082).

### In Vivo Comparison of OTL-38 Detection of Adenocarcinoma Compared With Squamous Cell Carcinoma

#### Our study population

There was a total of 326 patients included in our trial, with 211 who had lesions localized in vivo. Among those patients with lesions localized in vivo, there were 134 patients with AC, 9 with SCC, and 68 with other causes (metastases, carcinoid tumors, sarcomas). The mean age of patients who had AC was 66 (SD 8) years compared with 72 (SD 8) years in patients with SCC (*P* = .02). AC occurred more in female patients (*P* = .02), whereases SCC occurred equally in men and women (*P* > .05). All 15 patients who have SCC have a positive smoking history and a mean pack-year of 60.2 (SD 3,6), whereas 76% of patients with AC have a history of smoking and a mean pack-year of 29.3 (*P* = .02) (Supplemental Table 1, Supplemental Figure).

#### Pafolacianine preferentially labels adenocarcinoma compared with squamous cell carcinoma in vivo

On a univariate level, the ability of pafolacianine to localize the tumor in patients with AC compared with SCC had an odds ratio of 2.05 (*P* = .004). In the multivariate logistic regression model, AC was twice more likely to be localized than SCC, with an odds ratio for fluorescence of (AC/SCC) of 2.01 (*P* = .02).

#### Intraoperative molecular imaging with pafolacianine has a higher false negative rate with squamous cell carcinoma compared with adenocarcinoma

A nonfluorescent lung nodule that is cancer is more likely to be SCC (specificity of identifying SCC is 9.52% compared with 5.56% for AC) (Table, Supplemental Table 2).

**Table:** Measures of the Ability of Intraoperative Molecular Imaging With Pafolacianine to Identify Adenocarcinoma vs Squamous Cell Carcinoma

Histologic Type	Test			
	Sensitivity, %	Specificity, %	PPV, %	NPV, %
AC	88.6	5.56	71.%	15.4
SCC	86.7	9.52	5.4	92.3

AC, adenocarcinoma; NPV, negative predictive value; PPV, positive predictive value; SCC, squamous cell carcinoma.

## Comment

AC cell lines have a statistically significant higher fluorescence and FRα expression when compared with SCC. In our clinical trial, patients with AC were 2 times more likely to have lesions localized in vivo compared with patients with SCC. Finally, we found that a nonfluorescent lung nodule that is cancer is more likely to be SCC.

Previous studies have shown that FRα expression is associated with survival and is largely limited to AC with FRα expression in as much as 72% of AC compared with 13% SCC (*P* < .0001).^7^^,^^8^ The origins of these 2 tumor histologic types offer an explanation for this observation. AC is derived from type I and II pneumocytes that already express FRα. Meanwhile, SCC is derived from the more centrally located epithelium that does not express FRα. Conversely, FRβ is reported to be present on hematopoietic cells of myelogenous lineage. In lung cancer, these myelogenous lineage cells are TAMs.^9^ Similar to our findings, studies have shown that there is no significant difference in the expression of FRβ on the TAMs of AC and SCC. However, a higher FRβ expression in both tumor histologic types is associated with a worse prognosis.^9^ This is not the case with FRα. Hence, we can conclude that AC tends to express FRα more often. Meanwhile, increased FRβ expression correlates with a poorer prognosis in either cell line but is not differentially expressed in 1 tumor histologic type.

Our findings do not suggest that pafolacianine does not localize SCC. In fact, it has been shown that with IMI, we have improved detection of both AC and SCC compared with traditional white light.^2^ However, pafolacianine has a higher likelihood of localizing AC compared with SCC, and it has higher MFIs with AC. Knowing the patients’ tumor histologic type can aid in choosing the best contrast agent (among the many in the market). In fact, a recent study showed that evaluating FR expression is a low-cost tool for patient selection whereby patients with higher FR expression are more likely to fluoresce.^10^

In conclusion, tumor histology appears to play in role in IMI. With pafolacianine, patients with AC of the lung are more likely to fluoresce when compared with those with SCC. This difference is most likely attributed to increased folate expression in patients with AC. This study looked at tumor histology as an independent predictor of success of IMI in localizing disease. It introduced the concept of identifying patient-specific factors that can determine their candidacy for IMI. It also emphasized the importance of performing immunohistochemistry when selecting patients for IMI. Further studies are currently evaluating other aspects of a patient’s preoperative profile that can guide their personalized IMI techniques and dyes.
