# Supplementary material for: Histology and Lung Nodule Fluorescence in Intraoperative Molecular Imaging With Pafolacianine
Source: Ann Thorac Surg Short Rep. 2024 Mar 5;2(3):432–7. doi: 10.1016/j.atssr.2024.02.003 (PMC11708748; doi:10.1016/j.atssr.2024.02.003)
Supplement: Supplemental Table and Supplemental Figure [file mmc1.docx]

**Supplemental Figures and tables**

| Patient Characteristics | | AC | SCC | p-value |
| --- | --- | --- | --- | --- |
| Gender | Male | 54 | 8 | 0.04 |
|  | Female | 139 | 7 |  |
| Age at surgery (mean/SD) | | 66/8 | 72/8 | 0.02 |
| BMI (kg/m^2^) (mean/SD) | | 26.97/5.03 | 29.66/6.62 | 0.05 |
| Current Smoking | No | 169 | 11 | 0.11 |
|  | Yes | 23 | 4 |  |
| Smoking History | No | 46 | 0 | 0.02 |
|  | Yes | 147 | 15 |  |
| Smoking (pack-years) (mean/SD) | | 29.3/29.9 | 60.2/3.6 | <0.001 |
| NAT | No | 153 | 13 | 0.4 |
|  | Yes | 12 | 0 |  |
| Tumor Size (cm) (mean/SD) | | 2 (1.2) | 2.45 (1.6) | 0.3 |
| Distance From Pleura (cm) | | 0.37 (0.63) | 0.38 (0.63) | 0.9 |
| Differentiation | well | 24 | 0 | 0.07 |
|  | moderate | 100 | 9 |  |
|  | poorly | 38 | 6 |  |
|  | undifferentiated | 31 | 0 |  |
| PET | not avid | 34 | 0 | 0.06 |
|  | avid | 159 | 15 |  |

Table 1- Baseline characteristics of population

| IMI  Pathology | Detected by IMI | Not Detected by IMI |
| --- | --- | --- |
| AC | 171 | 22 |
| Not AC | 69 | 4 |
| SCC | 13 | 2 |
| Not SCC | 227 | 24 |

Table 2- Using IMI with pafolacianine to localize adenocarcinoma (AC) and squamous cell carcinoma (SCC) of the lung.


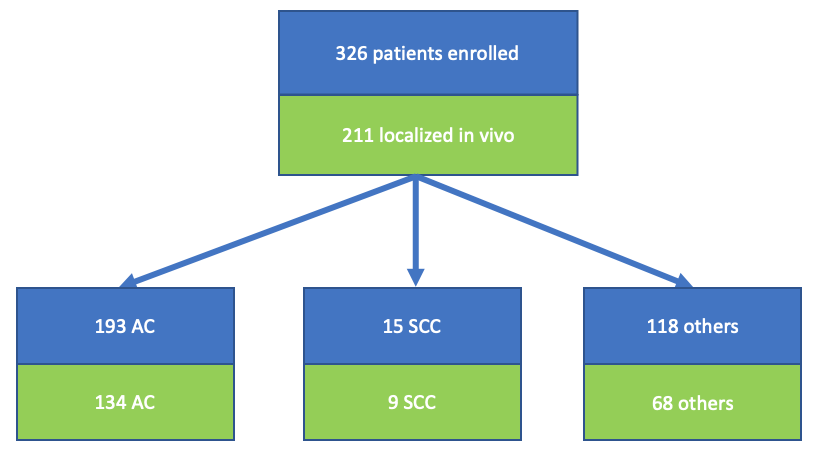


Figure – Histologic distribution of patients enrolled in trial. Blue indicates total number of patients and green indicates those identified using IMI

**Supplemental**

Inclusion and Exclusion Criteria of Clinical Trial
Patients were included if they were 18 years or older and had a primary diagnosis or high clinical suspicion of cancer warranting surgery based on their CT/PET scan. Exclusion criteria included allergy to any component of the dye, a positive pregnancy test, or impairment of renal or liver function.
Baseline characteristics such as sex, age, body mass index(BMI), smoking history, tumor size, tumor type, tumor differentiation, distance of tumor from surface, and PET avidity were collected.

Cell line maintenance

Cell lines were cultured on poly-L-lysine-coated glass coverslips in 6-well plates with folate-deficient DMEM/RPMI media supplemented with 10% FBS, L-glutamine and penicillin/streptomycin for 24 hr. Cells were incubated with 2 µM OTL38 for 2 hours at 37°C. For folate receptor inhibition experiments, cells underwent a 30-minute pretreatment with 200µM folate (Sigma-Aldrich, St. Louis, MO). Coverslips were removed from culture following OTL38 treatment, mounted on glass slides with ProLong Gold Antifade reagent with DAPI (Fisher Scientific, Waltham, MA), and imaged on a Leica DM6 B fluorescence microscope (Leica Microsystems, Wetzlar, Germany).
